# Complete Genome Sequence of *Bacillus pumilus* PDSLzg-1, a Hydrocarbon-Degrading Bacterium Isolated from Oil-Contaminated Soil in China

**DOI:** 10.1128/genomeA.01079-16

**Published:** 2016-10-06

**Authors:** Kun Hao, Hongna Li, Feng Li, Ping Guo

**Affiliations:** a Agricultural Clean Watershed Research Group, Institute of Environment and Sustainable Development in Agriculture, Chinese Academy of Agricultural Sciences, Beijing, People’s Republic of China; b State Key Laboratory for Biology of Plant Diseases and Insect Pests, Institute of Plant Protection, Chinese Academy of Agricultural Sciences, Beijing, People’s Republic of China

## Abstract

*Bacillus pumilus* strain PDSLzg-1, an efficient hydrocarbon-degrading bacterium, was isolated from oil-contaminated soil. Here, we present the complete sequence of its circular chromosome and circular plasmid. The genomic information is essential for the study of degradation of oil by *B. pumilus* PDSLzg-1.

## GENOME ANNOUNCEMENT

*Bacillus pumilus* strain PDSLzg-1 is a novel bacterium isolated from oil-contaminated soil of the Shengli oil field (Shandong, People’s Republic of China) in 2015. The bacterium is characterized as Gram-positive, aerobic, motile, rod-shaped, and forming endospores. According to 16s rRNA sequencing analysis, the bacterium is identified as a strain of *Bacillus pumilus*. This strain, designated PDSLzg-1, has the ability to effectively degrade petroleum hydrocarbons, especially C8 to C18 alkanes, and was shown to produce at least one bio-surfactant and may be of interest for an industrial application of oil pollution degradation.

*B. pumilus* PDSLzg-1 genomic DNA was isolated from overnight culture using a Wizard Genomic DNA purification kit (Promega). Total DNA obtained was subjected to quality control by agarose gel electrophoresis and quantified by Qubit. The genome of *B. pumilus* PDSLzg-1 was sequenced by single molecule real-time (SMRT) technology. Sequencing was performed at the Beijing Novogene Bioinformatics Technology Co., Ltd., producing 95,240 reads with 358× coverage. SMRT Analysis 2.3.0 was used to filter low-quality reads and the filtered reads were assembled to generate one contig without gaps. The genome of *B. pumilus* PDSLzg-1 had a 3,698,973-bp circular chromosome and an 11,801-bp circular plasmid with G+C contents of 41.96% and 39.36%, respectively. Gene prediction was performed on the *B. pumilus* PDSLzg-1 genome by GeneMarkS, resulting in the identification of 3,895 genes with an average length of 848 bp (1). 81 tRNA genes were predicted with tRNAscan-SE, 24 rRNA genes were predicted with rRNAmmer, and seven sRNAs were predicted by a BLAST search against the Rfam database (2–4). A whole-genome BLAST search (*E* value less than 1e−5, minimal alignment length percentage larger than 40%) was performed against KEGG, COG, nr, Swiss-Prot, GO, and TrEMBL (5–9). 3,689 (94.7%) genes were annotated to at least one database.

There were a group of genes in genome of *B. pumilus* strain PDSLzg-1 encoding alcohol dehydrogenase, aldehyde dehydrogenase, and monooxygenase, which are probably related to oil degradation (10, 11). This study presents the first fully sequenced and annotated hydrocarbon-degrading strain of *B. pumilus*, which is valuable for studying its evolution, hydrocarbon-degrading mechanism, and industrial applications.

### Accession number(s).

The complete annotated genome and plasmid sequences of *B. pumilus* strain PDSLzg-1 were deposited in GenBank under the accession numbers CP016784 and CP016785, respectively.
